# Phase 2 of CATALISE: a multinational and multidisciplinary Delphi consensus study of problems with language development: Terminology

**DOI:** 10.1111/jcpp.12721

**Published:** 2017-03-30

**Authors:** Dorothy V.M. Bishop, Margaret J. Snowling, Paul A. Thompson, Trisha Greenhalgh, and the CATALISE‐2 consortium, Catherine Adams, Lisa Archibald, Gillian Baird, Ann Bauer, Jude Bellair, Christopher Boyle, Elizabeth Brownlie, Glenn Carter, Becky Clark, Judy Clegg, Nancy Cohen, Gina Conti‐Ramsden, Julie Dockrell, Janet Dunn, Susan Ebbels, Aoife Gallagher, Simon Gibbs, Emma Gore‐Langton, Mandy Grist, Mary Hartshorne, Alison Hüneke, Marc Joanisse, Sally Kedge, Thomas Klee, Saloni Krishnan, Linda Lascelles, James Law, Laurence Leonard, Stephanie Lynham, Elina Mainela Arnold, Narad Mathura, Elspeth McCartney, Cristina McKean, Brigid McNeill, Angela Morgan, Carol‐Anne Murphy, Courtenay Norbury, Anne O'Hare, Janis Oram Cardy, Ciara O'Toole, Rhea Paul, Suzanne Purdy, Sean Redmond, Laida Restrepo, Mabel Rice, Vicky Slonims, Pamela Snow, Jane Speake, Sarah Spencer, Helen Stringer, Helen Tager‐Flusberg, Rosemary Tannock, Cate Taylor, Bruce Tomblin, Joanne Volden, Marleen Westerveld, Andrew house

**Affiliations:** ^1^ Department of Experimental Psychology University of Oxford Oxford UK; ^2^ Nuffield Department of Primary Care Health Sciences University of Oxford Oxford UK

**Keywords:** Developmental language disorder, specific language impairment, terminology, risk factors, definitions

## Abstract

**Background:**

Lack of agreement about criteria and terminology for children's language problems affects access to services as well as hindering research and practice. We report the second phase of a study using an online Delphi method to address these issues. In the first phase, we focused on criteria for language disorder. Here we consider terminology.

**Methods:**

The Delphi method is an iterative process in which an initial set of statements is rated by a panel of experts, who then have the opportunity to view anonymised ratings from other panel members. On this basis they can either revise their views or make a case for their position. The statements are then revised based on panel feedback, and again rated by and commented on by the panel. In this study, feedback from a second round was used to prepare a final set of statements in narrative form. The panel included 57 individuals representing a range of professions and nationalities.

**Results:**

We achieved at least 78% agreement for 19 of 21 statements within two rounds of ratings. These were collapsed into 12 statements for the final consensus reported here. The term ‘Language Disorder’ is recommended to refer to a profile of difficulties that causes functional impairment in everyday life and is associated with poor prognosis. The term, ‘Developmental Language Disorder’ (DLD) was endorsed for use when the language disorder was not associated with a known biomedical aetiology. It was also agreed that (a) presence of risk factors (neurobiological or environmental) does not preclude a diagnosis of DLD, (b) DLD can co‐occur with other neurodevelopmental disorders (e.g. ADHD) and (c) DLD does not require a mismatch between verbal and nonverbal ability.

**Conclusions:**

This Delphi exercise highlights reasons for disagreements about terminology for language disorders and proposes standard definitions and nomenclature.

## Introduction

Language problems are common in children, with prevalence estimates ranging from 3% to 7%, depending on age and definition (Norbury et al., 2016; Tomblin, Records et al., 1997; Weindrich, Jennen‐Steinmetz, Laucht, Esser, & Schmidt, 2000). In relation to their severity and prevalence, children's language problems receive considerably less research funding than other conditions such as attention deficit hyperactivity disorder (ADHD) or autism spectrum disorder (ASD), with which they frequently co‐occur (Bishop, 2010). The term Specific Language Impairment (SLI) has been widely used to refer to children whose language development is not following the usual course despite typical development in other areas. However, professionals and lay people alike appear to be far less familiar with SLI compared with dyslexia or autism (Kamhi, 2004). Of more concern, Ebbels (2014) described how use of the term SLI had become controversial, because it seemed not to reflect clinical realities and excluded many children from services.

Bishop, Snowling, Thompson, Greenhalgh and The CATALISE Consortium (2016) used an online version of the Delphi technique (Hasson, Keeney, & McKenna, 2000) with the aim of achieving consensus on these issues. Because of the complexity of the subject matter, we divided the task into two phases: the first, described by Bishop et al. (2016) focused on criteria for identifying significant language problems in children. Here we describe the second phase, where the same panel focused on the issue of terminology for children's language problems. Here we describe this second phase.

## Materials and methods

### Ethics approval

This research was approved by The Medical Sciences Interdisciplinary Research Ethics Committee, University of Oxford (approval number: MS‐IDREC‐C1‐2015‐061). Panel members gave written consent for their ratings to be used to derive a consensus statement.

### Delphi panel

We approached the same panel members who had formed part of the CATALISE consortium for our previous Delphi on criteria. As detailed by Bishop et al. (2016), we restricted consideration to English‐speaking countries, and there was a predominance of speech‐language therapists/pathologists (SLT/Ps). Of the original panel, two declined to take part in CATALISE‐2 for personal reasons, leaving a panel of 57 individuals, whose characteristics are shown in Table 1. Nine panel members had a close relative with impaired language development.

**Table 1:** Professional group and country^a^ of panel members

Profession	*N* and Country	Gender
Speech‐Language Therapist/Pathologist	31 (15 UK, 6 USA, 3 NZ, 3 Ire, 1 Can, 3 Aus)	6 M, 25 F
Joint SLT/SLP and Psychologist	7 (3 Can, 2 Aus, 2 UK)	1 M, 6 F
Psychologist/Educational Psychologist	8 (3 UK, 1 US, 3 Can, 1 Aus)	3 M, 5 F
Paediatrician	3 (3 UK)	1 M, 2 F
Psychiatrist	1 (1 Can)	1 F
Audiologist	1 (1 NZ)	1 F
Specialist teacher	2 (2 UK)	2 F
Charity representative	4 (4 UK)	4 F
Total	57	57

a Country where panel member was based at start of Delphi studies.

The first two authors (DVMB and MJS), both psychologists with considerable experience in the area of children's language problems, acted as moderators: they did not contribute rankings, but agreed on modifications to statements on the basis of feedback from the panel. The third author (PT) set up the online Delphi, controlled the anonymisation and analysed responses to produce reports for panel members. The fourth (TG), an expert in primary health care who was familiar with the Delphi method acted, as methodological advisor.

### Delphi consensus process

We started with a set of statements about terminology accompanied by a background document (Appendix S1) that put these in context. These were new statements that were different from those in the prior Delphi exercise on criteria, though they were informed by issues that arose in that study (Bishop et al., 2016). Panel members were asked to rate the statements on a 5‐point scale from 1 (strongly disagree) to 5 (strongly agree).

Participant responses to Round 1 were collated. The distribution of responses and associated anonymised comments were then fed back to all panel members and scrutinised by the moderators. One difference from our previous Delphi was that we held a 1‐day meeting to present and discuss preliminary results from CATALISE‐2 before proceeding to Round 2. All panel members were invited to this, as well as additional stakeholders. The meeting was attended by the first four authors and 22 of the CATALISE‐2 consortium, as well as 23 individuals representing a range of fields: eight from speech and language therapy, eight from psychology, one paediatrician, two representatives from charities, one expert in special educational needs, one geneticist, one general practitioner and one psychiatrist.

On the basis of ratings, qualitative comments and discussions at the meeting, the two moderators agreed on rewording of some items and revision of the background document. The set of items and background document used in Round 2 are shown in Appendix S2.

There is no agreed criterion for when a Delphi consensus is deemed adequate for an item – in the literature, values from 51% to 80% agreement have been used (Hasson et al., 2000). We aimed for 75% agreement as a reasonable goal.

After Round 2, the moderators made some further revisions to the statements to improve clarity and readability, to take into account specific comments provided by the panel, and to reconsider the two problematic items. Some statements with good agreement were consolidated to give a single longer statement (see Appendix S3), giving a total of 13 statements. A draft of the current paper, including finalised statements in the Results section, was circulated for comments and approval by the panel. Further revisions were made to address points raised by reviewers, including the dropping of one redundant statement, and the paper was again circulated to all panel members for comment. The current paper represents the final agreed version.

## Results and discussion

### Round 1

The response rate by panel members for Round 1 was 93%. Appendix S4 shows quantitative and qualitative responses to the Round 1 statements; a personalised copy of this report containing these data was sent to all panel members, showing how their own responses related to the distribution of responses from other (anonymised) panel members. The percentage agreement (combining strongly agree with agree) ranged from 30% to 98% for the 16 items, with a median value of 74%.

Kruskal–Wallis tests were conducted on each item to test whether agreement was related to either geographical location (six countries) or professional status (SLT/P vs. others), using a Bonferroni‐corrected *p*‐value of .001. None of these comparisons was statistically significant after correction for multiple comparisons. Given the small sample size, we cannot rule out an effect of these two factors on ratings, but the analysis offers some reassurance that responses did not simply pattern according to professional background or geographical location.

### Round 2

The response rate by panel members for Round 2 was 91%. Appendix S5 contains the data that were incorporated in a personalised report sent to all panel members for Round 2. The percentage agreement (combining ratings of strongly agree with agree) ranged from 46% to 98% across items, with a median value of 90%. Of the 21 items, 19 had agreement of 78% or more, which we regarded as adequate to accept that statement. Items 19 and 20, both concerned with terms for subtypes of language disorder, had 68% and 46% agreement respectively, indicating a need for further revision or omission.

### *Consensus statements*

In this section, we present final statements, with supplementary comments that reflect reasoning behind them, based on qualitative comments and discussion, supported by references where appropriate.

#### **Statement 1**

It is important that those working in the field of children's language problems use consistent terminology.

#### Supplementary comment

In Round 2, a version of this statement was included to orient the panel to our common goal. Although the terminology we propose is not novel, its adoption will require many people to change their practices, which will be difficult where there is a long‐standing preference for other terms. Nevertheless, panel members were strongly motivated to achieve a consensus, because the lack of consistency was recognised as a major problem for the field.

#### **Statement 2**

The term ‘language disorder’ is proposed for children who are likely to have language problems enduring into middle childhood and beyond, with a significant impact on everyday social interactions or educational progress.

#### Supplementary comment

This statement clarifies that prognosis should be a key factor in the definition of language disorder; that is, the term should include those with language problems that lead to significant functional impairments unlikely to resolve without specialist help. There is no sharp dividing line between language disorder and typical development, but we can use relevant information from longitudinal studies to help determine prognosis (see Statement 3).

Arguments for preferring the term ‘disorder’ to ‘impairment’ included the greater seriousness and importance associated with the term; consistency with other neurodevelopmental disorders (autism spectrum disorder, developmental coordination disorder, attention deficit hyperactivity disorder); and compatibility with the two main diagnostic systems, DSM‐5 (American Psychiatric Association, 2013) and ICD‐11 (Baird, personal communication).

Some panel members expressed concerns that the term ‘disorder’ had medical connotations and placed the problem ‘inside the child’, when it might be contextually dependent. It was thought to have negative associations for teachers and there were concerns that such a label could lead to low expectations. For this reason, our definition explicitly excludes children who have limited language skills because of lack of exposure to the language of instruction, or are likely to grow out of their problems. These children often benefit from educational interventions, and may require monitoring, but they should not be identified as language disordered.

Another objection to the term ‘disorder’ is that historically it has been interpreted as referring to a large mismatch between language and nonverbal ability. This interpretation has been widely adopted in some circles, but is discredited and is not part of our definition (Bishop et al., 2016) (see also Statement 8).

#### **Statement 3**

Research evidence indicates that predictors of poor prognosis vary with a child's age, but in general language problems that affect a range of skills are likely to persist.

#### Supplementary comment

Prognostic indicators will vary with age. Our focus here is on what we know about learning English.

##### Under 3 years

Prediction of outcome is particularly hard in children under 3 years of age. Many toddlers who have limited vocabulary at 18–24 months catch up, and despite much research, it can be difficult to identify which late talkers are likely to have long‐term problems (Reilly et al., 2010). Children who fail to combine words at 24 months appear to have worse outcomes than those who do not produce any words at 15 months, though this is still a far from perfect predictor (Rudolph & Leonard, 2016). Prognosis is also poorer for children with comprehension problems, those who do not communicate via gesture (Ellis & Thal, 2008), or do not imitate body movements (Dohmen, Bishop, Chiat, & Roy, 2016). Roy and Chiat (2014) administered a preschool measure of social responsiveness and joint attention to 2‐ to 4‐year olds referred for speech‐language therapy, and found it was predictive of persisting problems, and indicative of social communication problems at 9 years. A positive family history of language or literacy problems is an additional risk factor (Rudolph & Leonard, 2016; Zambrana, Pons, Eadie, & Ystrom, 2014). Overall, however, the prediction from late language emergence to subsequent language disorder at school age is surprisingly weak: in part because many late talkers catch up but also because some school‐aged children with language disorder were not late to talk (Snowling, Duff, Nash, & Hulme, 2016; Zambrana et al., 2014).

##### Three to four years

Prediction improves as children grow older; in 4‐year olds, the greater the number of areas of language functioning that is impaired, the higher the likelihood that the problems will persist into school age (Bishop & Edmundson, 1987). Note that this finding contradicts the idea that intervention should be focused on children with a ‘spiky’ language profile rather than a more even pattern of impairment. When individual language tests are considered, sentence repetition has been identified as a relatively good marker for predicting outcomes (Everitt, Hannaford, & Conti‐Ramsden, 2013).

In contrast, there is generally a good prognosis for preschoolers whose problems are restricted to expressive phonology (Beitchman, Wilson, Brownlie, Walters et al., 1996; Bishop & Adams, 1990).

##### Five years and over

Language problems that are still evident at 5 years and over are likely to persist (Stothard, Snowling, Bishop, Chipchase, & Kaplan, 1998). Children who start school with oral language problems are at risk of reading problems and poor academic attainment (Bishop & Adams, 1990; Catts, Fey, Tomblin, & Zhang, 2002; Thompson et al., 2015) with little evidence that the language gap closes over time (Rice & Hoffman, 2015). Prognosis appears particularly poor when receptive language is impaired (Beitchman, Wilson, Brownlie, Walters, & Lancee, 1996; Clark et al., 2007), and when nonverbal ability is relatively low (Catts et al., 2002; Johnson, Beitchman, & Brownlie, 2010; Rice & Hoffman, 2015).

##### Family factors

There has been some debate over the predictive value of family factors. As noted above, several studies found that a positive family history of language problems is a predictor (albeit weak) of persisting problems in late talkers, and family history is also associated with poor literacy outcomes (Snowling & Melby‐Lervåg, 2016). It is less clear whether social background is independently predictive, once other risk factors have been taken into account (Botting, Faragher, Simkin, Knox, & Conti‐Ramsden, 2001).

For further discussion of the range of language skills under consideration, see Statement 11.

#### **Statement 4**

Some children may have language needs because their first or home language differs from the local language, and they have had insufficient exposure to the language used by the school or community to be fully fluent in it. This should not be regarded as language disorder, unless there is evidence that the child does not have age‐appropriate skills in any language.

#### Supplementary comment

This statement makes it clear that a low score on a language test does not necessarily mean that a child has any kind of disorder. It is important to consider whether the child has adequate proficiency in any language. In general, multilingualism does not lead to language problems (Paradis, 2016), but where there has been limited experience with the language used at school, the child may require extra help (Cattani et al., 2014). This also applies to hearing‐impaired children whose native language is a signed language. In practice, however, for many languages, we lack suitable (normed) assessments (Jordaan, 2008).

#### **Statement 5**

Rather than using exclusionary criteria in the definition of language disorder, we draw a threefold distinction between differentiating conditions, risk factors and co‐occurring conditions.

#### Supplementary comment

Use (and misuse) of exclusionary factors in definitions of language disorder was a major issue leading to dissatisfaction with terminology in this field. Panel members were concerned that, instead of being used for diagnostic differentiation, exclusionary criteria were sometimes interpreted as criteria for denying services to children. On the other hand, grouping together all children with a language problem, regardless of cause, and without regard to type of intervention required, would, in many contexts, be counterproductive.

Statements 6–10 explain how we draw the distinction between differentiating conditions, risk factors and co‐occurring conditions.

#### **Statement 6**

Differentiating conditions are biomedical conditions in which language disorder occurs as part of a more complex pattern of impairments. This may indicate a specific intervention pathway. We recommend referring to ‘Language disorder associated with X’, where X is the differentiating condition, as specified above.

#### Supplementary comment

Differentiating conditions include brain injury, acquired epileptic aphasia in childhood, certain neurodegenerative conditions, cerebral palsy and oral language limitations associated with sensori‐neural hearing loss (Tomblin et al., 2015) as well as genetic conditions such as Down syndrome. We also include here children with autism spectrum disorder (ASD) and/or intellectual disability (Harris, 2013) because these conditions are commonly linked to genetic or neurological causes (Fitzgerald et al., 2015; Shevell, Majnemer, Rosenbaum, & Abrahamowicz, 2001), with the numbers of known aetiology increasing with advances in genetic methods (Bourgeron, 2015; Fitzgerald et al., 2015; Shevell et al., 2001).

These are all cases where an association between a biomedical condition and language disorder is commonly seen. In such cases, the child requires support for the language problems, but the intervention pathway will need to take into account the distinctive features of the biomedical condition. It should be noted, however, that there is little research directly comparing language intervention approaches across conditions, so this inference is based on clinical judgement rather than research evidence.

#### **Statement 7**

The term Developmental Language Disorder (DLD) is proposed to refer to cases of language disorder with no known differentiating condition (as defined in Statement 6). Distinguishing these cases is important when doing research on aetiology, and is likely also to have implications for prognosis and intervention.

#### Supplementary comment

The term ‘Developmental Language Disorder’ is consistent with ICD‐11 (Baird, personal communication), though our definition does not include any nonverbal ability criteria.

‘Developmental’ in this context refers to the fact that the condition emerges in the course of development, rather than being acquired or associated with a known biomedical cause. Although many panel members endorsed it, some objections to the term ‘developmental’ were encountered. It was noted that ‘developmental’ can become less useful, or even confusing, as individuals grow older. One proposed solution was to drop the ‘developmental’ part of the term in adulthood – this is how this issue is typically handled in the case of (developmental) dyslexia, where affected adults usually refer to themselves as ‘dyslexic’. Some panel members noted specific meanings of ‘developmental’ that were not intended: for example, that this was something that the child might ‘grow out of’, or – quite the converse – that a developmental problem meant that the child would be unable to develop language. It was also suggested that this term might be hard for parents to understand – though similar objections were made for other alternatives that were offered, namely ‘primary’ and ‘specific’ language disorder.

#### **Statement 8**

A child with a language disorder may have a low level of nonverbal ability. This does not preclude a diagnosis of DLD.

#### Supplementary comment

It is important to recognise that language can be selectively impaired in a child with normal nonverbal ability, but this statement confirms that a large discrepancy between nonverbal and verbal ability is not *required* for a diagnosis of DLD. In practice, this means that children with low nonverbal ability who do not meet criteria for intellectual disability (Harris, 2013) can be included as cases of DLD.

#### **Statement 9**

Co‐occurring disorders are impairments in cognitive, sensori‐motor or behavioural domains that can co‐occur with DLD and may affect pattern of impairment and response to intervention, but whose causal relation to language problems is unclear. These include attentional problems (ADHD), motor problems (developmental coordination disorder or DCD), reading and spelling problems (developmental dyslexia), speech problems, limitations of adaptive behaviour and/or behavioural, and emotional disorders.

#### Supplementary comment

The terminology used for neurodevelopmental disorders can create the impression that there is a set of distinct conditions, but the reality is that many children have a mixture of problems. Indeed, the same problems may be labelled differently depending on the professional the child sees. For example, the same child may be regarded as having DLD by a SLT/P, dyslexia by a teacher, auditory processing disorder by an audiologist, or ADHD by a paediatrician. Given our focus on DLD, our aim with this statement was to make it clear that presence of another neurodevelopmental diagnosis does not preclude DLD.

Some panel members noted that a case could be made for including ASD as a co‐occurring disorder, rather than a differentiating factor. One reason for keeping it as a differentiating factor is that a substantial minority of children with ASD have a clear genetic aetiology: changes in chromosomes, copy number variants or specific mutations are estimated as accounting for around 25% of cases (Bourgeron, 2015), a figure likely to increase with advances in genetic methods. This is in contrast with the other neurodevelopmental disorders listed here, where, although there is evidence for heritability, the aetiology appears to be complex and multifactorial (see e.g. Bishop (2015) on dyslexia). In addition, communication problems are a core diagnostic feature of ASD, albeit with wide variation in the severity and nature of the language problems (Williams, Botting, & Boucher, 2008). Finally, the co‐occurring social and behavioural difficulties suggest the need for a distinctive intervention approach for ASD.

There was discussion about including auditory processing disorder (APD) as a co‐occurring condition. This category is controversial (Moore, 2006), but this should not lead to it being ignored. Children who are given this diagnosis often have co‐occurring language problems which require expert evaluation (Dawes & Bishop, 2009; Sharma, Purdy, & Kelly, 2009).

Some panel members noted that relatively pure cases without co‐occurring problems might be more common in epidemiological than in clinical samples. However, that this may in part reflect the criteria used to define cases in epidemiological studies, who may not be screened for difficulties in domains beyond language and IQ. A focus on ‘pure’ cases has been traditional in research settings, because it can clarify which features of a disorder are specific to language. However, this can make it difficult to generalise research findings to many children seen in clinical settings, where co‐occurring conditions are more commonly observed. Most panel members agreed that the term DLD should apply whether or not co‐occurring problems are documented.

#### **Statement 10**

Risk factors are biological or environmental factors that are statistically associated with language disorder, but whose causal relationship to the language problem is unclear or partial. Risk factors do not exclude a diagnosis of DLD.

#### Supplementary comment

These are factors that are not robust predictors of individual children's language status or outcome, but which are more common in children with language disorders than typically developing children (Zubrick, Taylor, & Christensen, 2015). A systematic review found that commonly documented risk factors include a family history of language disorders or dyslexia, being male, being a younger sibling in a large family and fewer years of parental education (Rudolph, 2016). Prenatal/perinatal problems do not seem to be an important risk factor for language disorders (Tomblin, Smith, & Zhang, 1997; Whitehouse, Shelton, Ing, & Newnham, 2014).

It is important to note that associated risk factors may differ depending on the age of the child, and whether epidemiological or clinical samples are considered.

#### **Statement 11**

Developmental language disorder is a heterogeneous category that encompasses a wide range of problems. Nevertheless, it can be helpful for clinicians to pinpoint the principal areas for intervention, and researchers may decide to focus on children with specific characteristics to define more homogeneous samples for study. We suggest here some guidelines for more in‐depth analysis of language problems.

#### Supplementary comment

The panel members did not reach good agreement on terminology for subgroups, and this may reflect the fact that, although attempts have been made to develop a classification of subtypes, these have not in general been validated as categories that are stable over time (Conti‐Ramsden & Botting, 1999). The traditional distinction used in DSM, between receptive and expressive language disorder, is rather gross, and fails to indicate which aspects of language are proving problematic. We have therefore opted for an approach that uses specifiers indicating the principal dimensions of language difficulty, with a recommendation that assessment focus on identifying which areas are most impaired. We outline these briefly below. Note: our focus here is on oral rather than written language, though reading and writing are commonly affected in DLD.

##### Phonology

Phonology is the branch of linguistics concerned with the organisation of speech sounds into categories. Different languages use different articulatory features to signal contrasts in meaning, and when learning language, the child has to learn which features to ignore and which to focus on (Kuhl, 2004).

In both research and clinical practice, most emphasis has been placed on expressive phonological problems: difficulties with speech production that are linguistic in origin, rather than due to motor impairment or physical abnormality of the articulators. This kind of problem is identified when a child fails to make a speech distinction between sounds that are used to contrast meaning in the language being learned, as when a child says ‘tea’ rather than ‘key’, substituting/t/for/k/. Phonological errors of this kind are common in early development, but can persist and, when numerous, impair intelligibility of speech. Phonological problems in preschoolers that are not accompanied by other language problems are a relatively common reason for referral to a SLT/P and often respond well to specialist intervention (Law, Garrett, & Nye, 2003). Thus, they would not meet our criteria for DLD because the prognosis is good. The more general term ‘Speech Sound Disorder’ (SSD) can be used for such cases: this is an umbrella term that also includes problems with speech production that have motor or physical origins, or involve misarticulations such as a lisp, where a sound is produced in a distorted way without losing the contrast with other sounds. The classification of and terminology for disorders of speech sound production is a subject of considerable debate (Waring & Knight, 2013). In practice, even for those with specialist skills, it is not always easy to distinguish between phonological disorders and other types of speech production problem.

Where phonological problems continue beyond 5 years of age it is important to assess the child's broader language skills, as persisting phonological difficulties are usually accompanied by other language problems and have a poorer prognosis (Bird, Bishop, & Freeman, 1995; Bishop & Edmundson, 1987; Hayiou‐Thomas, Carroll, Leavett, Hulme, & Snowling, 2017), so would merit a diagnosis of DLD. Where the child has a mixture of language disorder and motor or structural problems with speech production, a dual diagnosis of DLD with SSD is appropriate.

Some children have impairment affecting phonological awareness, that is they have difficulty explicitly categorising and manipulating the sounds of language. For instance, they may be unable to identify the three phonemes constituting the word ‘cat’, or to recognise that ‘cat’ and ‘car’ begin with the same phoneme. Phonological awareness has been studied extensively in children with reading disability, where it is commonly impaired, even in children with normal speech production. Although phonological awareness is often deficient in children with DLD, we would not diagnose DLD on the basis of poor phonological awareness alone, because it is a metalinguistic skill that can be as much a consequence as a cause of literacy problems (Wimmer, Landerl, Linortner, & Hummer, 1991).

##### Syntax

A considerable body of research has focused on documenting syntactic impairments in children with DLD (Van der Lely, 2005). Expressive problems with morpho‐syntax are of particular theoretical interest, and there have been contrasting attempts to account for them in terms of linguistic and processing theories (Leonard, 2014). Receptive language impairments affecting syntax can also occur, with children failing to interpret meaning conveyed by grammatical contrasts (Hsu & Bishop, 2014), or showing problems in distinguishing grammatical from ungrammatical sentence forms (Rice, Wexler, & Redmond, 1999).

##### Word finding and semantics

Some children struggle to produce words despite having some knowledge of their meaning – these are known as ‘word finding difficulties’ (Messer & Dockrell, 2006). Others have limited knowledge of word meanings – a problem that comes under the domain of lexical semantics. The child may be poor at understanding multiple word meanings and/or use a restricted vocabulary. The latter problem has been particularly noted in verb use, where the term ‘general all‐purpose verbs’ has been coined to describe this phenomenon (Kambanaros & Grohmann, 2015; Rice & Bode, 1993). Semantic impairments also encompass problems with expressing or understanding meaning from word combinations; for example, understanding the scope of the quantifier (all/none) in sentences such as ‘all the pens are in the boxes’ or ‘none of the pens are in the boxes’ (Katsos, Roqueta, Estevan, & Cummins, 2011).

##### Pragmatics/language use

Pragmatic difficulties affect the appropriate production or comprehension of language in a given context. They include such characteristics as providing too much or too little information to a conversational partner, insensitivity to social cues in conversation, being over‐literal in comprehension and having difficulty understanding figurative language (Adams, 2002). Prosodic abnormalities, in which cues such as intonation and stress are used idiosyncratically, so speech sounds robotic, stereotyped or otherwise atypical to the context, can also be disruptive to social communication. These difficulties are hallmarks of the communicative problems seen in ASD, but are also found in children who do not meet criteria for autism.

Specific terminology has been proposed for nonautistic children with pragmatic impairments. In ICD‐11, the term pragmatic language impairment is used as a descriptive qualifier within DLD. In DSM‐5, a new category of social (pragmatic) communication disorder (SPCD) has been introduced – see Baird and Norbury (2016).

We considered adopting the DSM‐5 term in CATALISE, but decided against this for several reasons. First, in DSM‐5, SPCD is seen as a new category of neurodevelopmental disorder, whereas we regard pragmatics as part of language, and hence pragmatic impairment as a type of language disorder. Second, the label SPCD emphasises social communication, rather than language; in contrast, our focus is on linguistic problems.

Interventions are being developed that address linguistic as well as social aspects of such communication problems (Adams, 2008), and a focus on pragmatic language as a feature of DLD should help direct children to appropriate intervention.

##### Discourse

In contexts such as narrative, children must learn to process sequences of utterances, so that they form a coherent whole. Children who lack this ability may produce sequences of utterances that appear disconnected and hard to follow. They may also experience comprehension failure if they interpret one sentence at a time, without drawing the necessary inferences to link them together (Karasinski & Weismer, 2010).

##### Verbal learning and memory

The research literature has shown that many children with DLD have problems in retaining sequences of sounds or words over a short delay (verbal short‐term memory), learning associations between words and meaning, or learning statistical patterns in sequential input (Archibald & Gathercole, 2006; Bishop, North, & Donlan, 1996; Campbell, Dollaghan, Needleman, & Janosky, 1997; Conti‐Ramsden, 2003; Ellis Weismer, 1996; Gillam, Cowan, & Day, 1995; Leonard 2007; Lum, Conti‐Ramsden, Page, & Ullman, 2012; Lum & Zarafa, 2010; Montgomery, 2002). Their language limitations are different from those due to poor hearing or auditory discrimination, or to lack of knowledge due to unfamiliarity with the ambient language.

**Figure 1:**
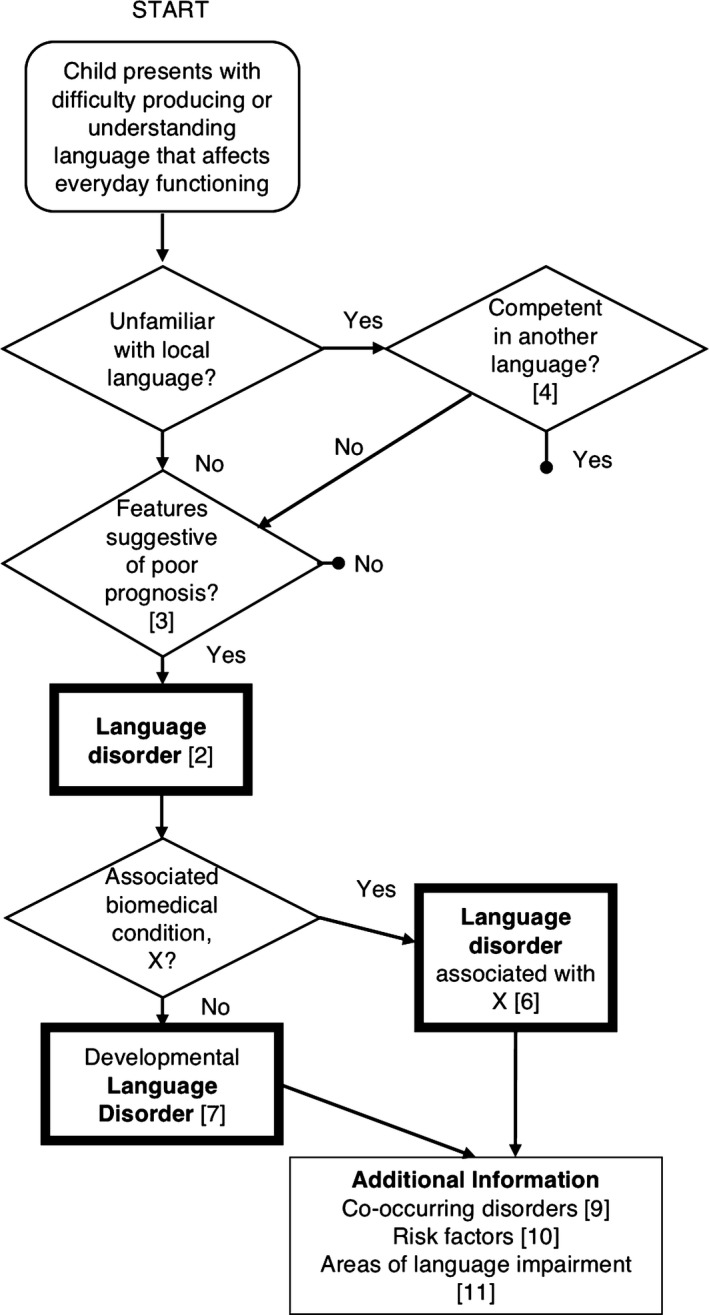
Flow chart illustrating pathways to diagnosis of language disorder. Numbers in square brackets refer to Statements in the Results section

Statements 2–11 are synthesised in Figure 1.

#### **Statement 12**

It can be useful to have a superordinate category for policymakers, because the number of children with specific needs in the domain of speech, language and communication has resource implications. The term Speech, Language and Communication Needs (SLCN), already in use in educational services in the United Kingdom, is recommended for this purpose.

#### Supplementary comment

DLD can be viewed as a subset within a broad category that covers the whole range of problems affecting speech, language and communication, regardless of the type of problem or putative aetiology.

**Figure 2:**
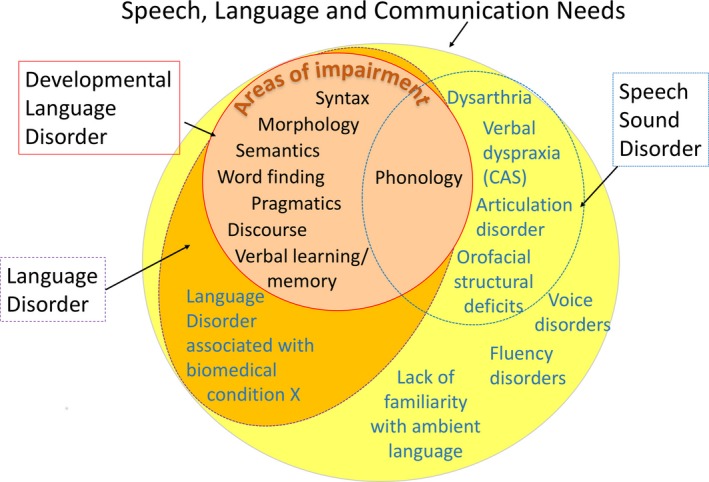
Venn diagram illustrating relationship between different diagnostic terms. DLD is nested within the broader SLCN category

As shown in Figure 2, this is a very broad category that encompasses children with DLD (as defined above), and also includes cases where problems have a clear physical basis (e.g. dysarthria), or affect speech fluency or voice. Also included here are children who have needs due to limited familiarity with the language used in the classroom, and those who have communication difficulties as part of other differentiating conditions.

It is not anticipated that this terminology will be useful for those doing research on the nature or causes of language disorders, nor will it be helpful in explaining a child's difficulties to parents or in determining a treatment pathway. It could, however, serve a purpose for those who need to plan services, who may need to estimate how many children are likely to require additional support and to bridge across professional divides (McKean et al., 2017). In addition, it recognises children who have language needs that may require extra help or accommodations in the classroom, even if they do not have a language disorder. These would include those who are shown in pathways terminating in a bullet in the flow chart in Figure 1, that is children with milder difficulties who should respond well to classroom modification, children with hearing loss who use sign language or children who have had limited exposure to the ambient language.

## General discussion

Despite the geographical and professional diversity of the panel, there were some points of broad agreement, as follows: first, some children have language problems that are severe and persistent enough to create long‐term functional challenges, in daily communication and/or educational attainment; second, there is no clear dividing line between normality and disorder; third, within the domain of language, children's problems do not neatly segregate into subtypes, and there may be overlap between problems in speech, language and communication.

A complicating factor in the nosology of language disorders is that it has in the past been based on information from a mixture of different levels of description: information about the severity and type of presenting problems with language; co‐occurring problems in nonlanguage domains, such as nonverbal ability, social interaction or attention; and putative biological and environmental causes, such as brain damage, a genetic syndrome or social disadvantage. This approach implies that the constellation of verbal and nonverbal skills will map onto natural subtypes with distinct causes, such that we can use the linguistic, cognitive and behavioural profile to distinguish the child whose language problems have environmental or genetic origins. However, this approach has not worked. As research has progressed, it has become evident that causes of language disorders are complex and multifactorial, and there is no neat one‐to‐one mapping between aetiology and phenotype.

In many ways, the results of this consensus exercise may seem unsurprising. The principal recommended term, DLD, has a long history in the field, and is compatible with planned usage in ICD‐11 and close to the term (Language Disorder) used in DSM‐5. It was one of four possible terms considered in Bishop's (2014) original review of terminology, and already had reasonable representation in a Google Scholar search. For many of those working in this area, however, this represents quite a radical departure from previous practice. The term Specific Language Impairment, which was the most frequent in the research literature, was the subject of substantial disagreement among the panel, with strong arguments being put forward both for its retention and its rejection. Ultimately, the decision was made to reject the term. A major drawback of this decision is that it creates a discontinuity with prior literature, which could affect future meta‐analyses and systematic reviews. On balance, however, it was concluded that the term ‘specific’ had connotations that were misleading and confusing and that, rather than redefining the term, it would be better to abolish it.

There are other aspects of terminology where the Delphi process exposed points of disagreement, but also clarified reasons for these and so allowed us to identify ways forward. Discussions about the term ‘disorder’ revealed principled objections by those who were concerned about medicalisation of normal developmental variation. At the same time, concerns were expressed that other terminology might trivialise the challenges experienced by children who had persistent problems that interfered with their social and educational development. The solution we adopted was to retain ‘disorder’ but define it in a way that required functional problems with a poor prognosis. This may seem a small change, but it does have major implications. In particular, it cautions against defining language disorder solely in terms of statistical cut‐offs on language tests. Note also that we reject any attempt to use discrepancy scores to draw a distinction between ‘disorder’ and ‘delay’: the term ‘language delay’ was widely rejected by our panel members as confusing and illogical.

The main challenge facing those attempting to use the concept of language disorder that we advocate is that there are few valid assessments of functional language and relatively limited evidence regarding prognostic indicators. More longitudinal research is needed, using designs that allow us to predict individual outcomes rather than just characterise group averages.

A further case where the Delphi process helped identify sticking points was the treatment of ‘exclusionary factors’. We hope that our distinction between differentiating conditions, risk factors and co‐occurring disorders will be helpful here. Only differentiating conditions, which correspond to biomedical disorders that are clearly associated with language problems, are distinguished diagnostically from DLD. Risk factors and co‐occurring disorders are noted but do not preclude a diagnosis of DLD. This contrasts with prior practice in some quarters, where a child's social background or presence of problems in other developmental areas could leave a child without a diagnosis, and hence without access to support.

Finally, although it was generally agreed that there is considerable heterogeneity in children with DLD, we failed to reach consensus about possible terminology for linguistic subtypes of DLD. It is possible that as research advances the situation may change, but another possibility is that it is a consequence of the phenomenon of interest: quite simply, children with DLD do not neatly divide into subtypes along linguistic lines. It is likely that there is substantial aetiological as well as linguistic heterogeneity, just as has been found for the related conditions of ASD (Coe, Girirajan, & Eichler, 2012) and developmental dyslexia (Raskind, Peter, Richards, Eckert, & Berninger, 2012). In addition, the boundaries between DLD and other neurodevelopmental disorders are not clearcut (Bishop & Rutter, 2008). In our current state of knowledge, we propose that the appropriate course of action is to document the heterogeneity rather than attempting to apply a categorical nosology that fails to accommodate a large proportion of children.

An obvious limitation of this study is that we restricted our focus to the English language because of the difficulties of devising terms that would be applicable across different language and cultures. We recommend the use of the Delphi method to researchers working with language disorders in other languages, as a good way to achieve better consensus.

As with our previous Delphi study, this exercise has revealed the urgent need for further research on children's language disorders, including studies on intervention, models of service delivery, epidemiology, prognosis, linguistic profiles and functional limitations over time. We hope that by clarifying terminology in this area we will also make it easier to raise awareness of children's language problems.

**Key points**

- Some children have problems with language development that cause significant interference with everyday life or educational progress. Terminology for describing such problems has been inconsistent, hampering communication, leading to inequity over access to services and confusion in synthesising research.
- A group of experts representing a range of professions and English‐speaking countries using the Delphi method, came to a consensus that ‘Developmental Language Disorder’ (DLD) is the preferred term for language problems that are severe enough to interfere with daily life, have a poor prognosis and are not associated with a clear biomedical aetiology.
- We replace the traditional exclusionary criteria in the definition of language disorder, with a threefold distinction between differentiating conditions, risk factors and co‐occurring conditions.
- We provide guidelines about terminology in this area that can be used in clinical and research contexts.

## Supporting information


**Appendix S1.** Background document, with the statements for round 1.

**Appendix S2.** Background document, with the statements for round 2.

**Appendix S3.** Relationship between Round 2 statements and final statements reported in Results section.

**Appendix S4.** Report showing quantitative and qualitative responses to Round 1 statements.

**Appendix S5.** Report showing quantitative and qualitative responses to Round 2 statements.
