# Supplementary material for: Phase 2 of CATALISE: a multinational and multidisciplinary Delphi consensus study of problems with language development: Terminology
Source: J Child Psychol Psychiatry. 2017 Mar 30;58(10):1068–80. doi: 10.1111/jcpp.12721 (PMC5638113; doi:10.1111/jcpp.12721)
Supplement: Supplementary file 1 — Appendix S1. Background document, with the statements for round 1. [file JCPP-58-1068-s001.docx]

**CATALISE PHASE 2: Terminology**

**Background preamble**

CATALISE ONE converged so as to give us three strands that need to be considered when identifying language problems, as shown in panels A, B and C.
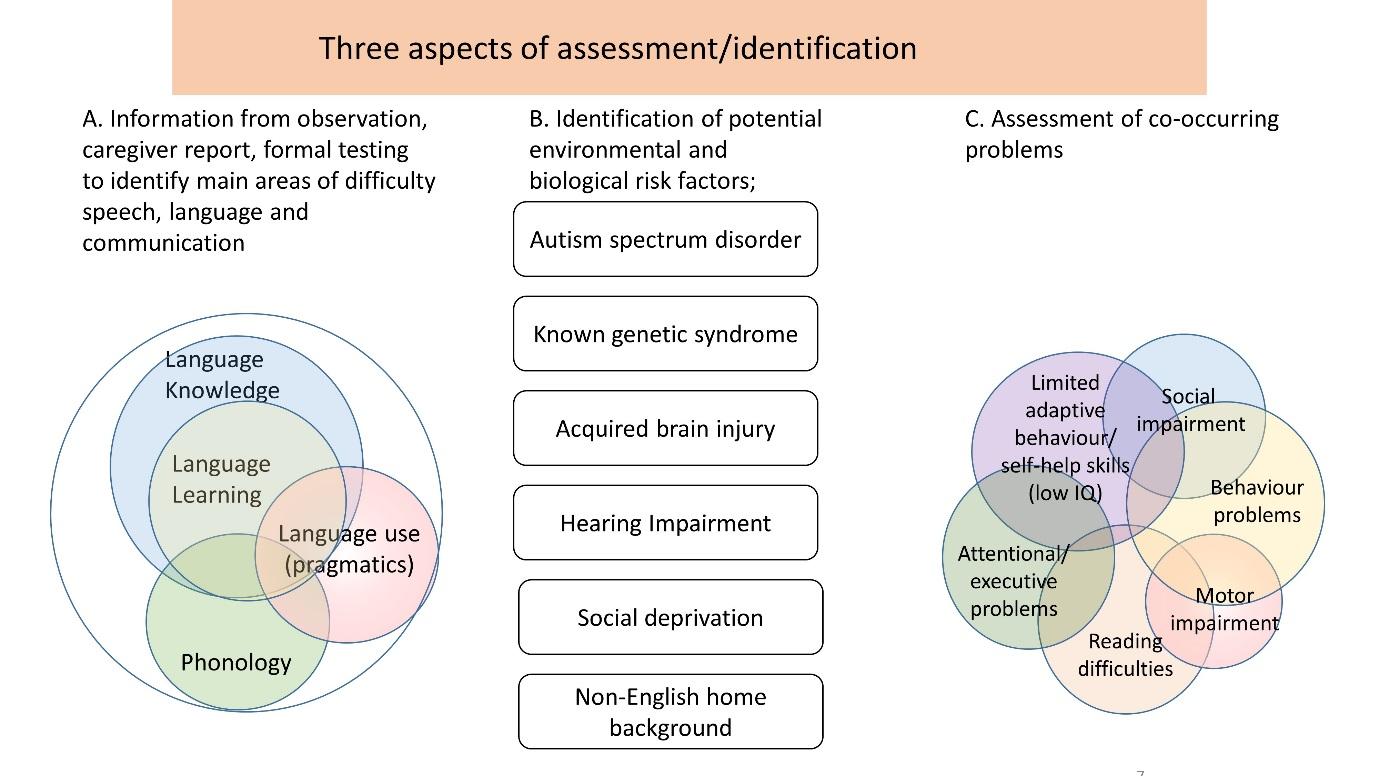


Please rate the following statements, which are intended to form the basis for recommendations on terminology.

**I. General principles**

1. The principal basis for terminology adopted in this field should be panel A observations (i.e., the nature and extent of language difficulties), with information from panels B and C playing a supplementary role.

2. Terminology needs to be flexible in order to be suitable for different needs in terms of policy, education, clinical practice and research, but within each of those domains, we should aim for clarity and consistency.

**II. Terminology for types of language impairment**

**Figure 1**


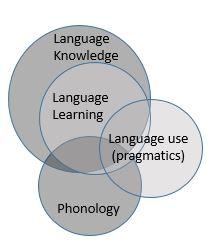


3. The overlapping subsets shown in Figure 1 (from panel A) all refer to problems with language. The generic term **Language Impairment** is recommended for situations where it is useful to adopt a broad category, where there is difficulty differentiating types of language difficulty, or where difficulties affect many domains of language.

N.B. if you prefer an alternative to 'impairment', such as 'disorder' or 'disability', please use the comments to specify this. Please note too that the use of specifier such as 'primary' or 'specific' with the term Language Impairment is covered in items 12-15 below).

4. More specific terms may be useful for specialist SALTS/SLPs or for researchers, to indicate specific types of problem. This terminology is likely to change as knowledge advances.

**Figure 2**


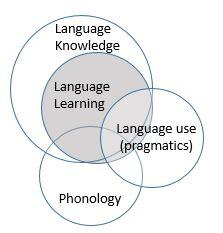


5. The grey area in Figure 2 shows cases where the main problems are in the language learning domain (as evidence by tests of memory and learning and/or lack of response to intervention). For such cases we propose the term **Language Learning Impairment**

**Figure 3**


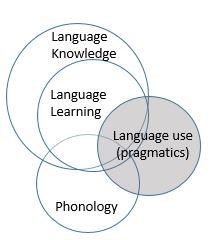


6. For children whose main problems are with pragmatics/social communication (shaded area in figure 3), and who do not meet diagnostic criteria for Autism Spectrum Disorder, we propose the term **Social Communication Impairment.**

(N.B. If you prefer the alternative term Pragmatic Language Impairment please note this in the comments. We avoided it here because it would create confusion if the acronym PLI is also used for primary language impairment – see item 13).

**Figure 4**


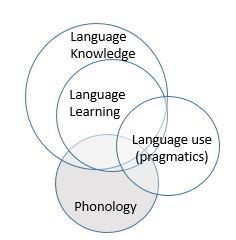


7. For children whose main problems are with phonology (shaded area in figure 4), we propose the term **Phonological Impairment.** This excludes cases where there is a structural or motor cause of a speech difficulty.

8. For policymakers, it can be useful to have a broad category that encompasses the full range of language problems in Panel A, and also includes cases where problems have a clear physical basis (e.g. dysarthria), affect speech fluency, voice or auditory perception, or involve other aspects of communication (e.g. use of augmentative or assistive technologies). The term **Speech, Language and Communication Needs (SLCN**), already in use in educational services in the UK, is recommended for this purpose.

**III. Risk factors**

As shown in panel B, risk factors include Autism spectrum disorder, known genetic syndromes, acquired brain injury, hearing impairment, social deprivation and non-English home background.

9. Where a risk factor, X, is present, the impairment can be described as **Secondary to X**, provided that the language difficulties are consistent with what is known about the impact of X.

10. The mere presence of a risk factor does not necessarily mean it explains the child's difficulties. For instance, a child with hearing loss may have language difficulties affecting mastery of sign language; a child with English as an Additional Language may show evidence of language learning difficulties rather than just limited language knowledge.

11. In clinical contexts, there does not seem any justification for allocating services according to whether language difficulties are primary or secondary.

12. In some contexts (e.g. research on aetiology), one may wish to restrict consideration to cases who do not have any known risk factors, and in other contexts it may be useful to make explicit that there is a lack of risk factors.

Please use the next 3 items to indicate your views on different options for terminology for such cases (and use comments to expand on your rating or make additional suggestions).

13. The preferred term for cases of language impairment that are not secondary to specific risk factors is **Primary Language Impairment** (see also items 14 and 15).

14. The preferred term for cases of language impairment that are not secondary to specific risk factors is **Specific Language Impairment** (see also items 13 and 15).

15. The preferred term for cases of language impairment that are not secondary to specific risk factors is **Developmental Language Impairment** (see also items 13 and 14).

**IV. Additional developmental difficulties**

16. Developmental difficulties in non-language domains are common and are likely to affect prognosis. Additional difficulties in the areas shown in panel C should be noted but not used as exclusionary criteria.
