# Supplementary material for: Phase 2 of CATALISE: a multinational and multidisciplinary Delphi consensus study of problems with language development: Terminology
Source: J Child Psychol Psychiatry. 2017 Mar 30;58(10):1068–80. doi: 10.1111/jcpp.12721 (PMC5638113; doi:10.1111/jcpp.12721)
Supplement: Supplementary file 2 — Appendix S2. Background document, with the statements for round 2. [file JCPP-58-1068-s002.docx]

**Explanatory Document**

**CATALISE PHASE 2: Terminology**

**Importance of achieving consensus**

***Statement 1****: Achieving agreed terminology for children's language difficulties is a high priority for the field, even if we cannot achieve perfect consensus.*

We added this statement at the suggestion of the authors of the Dutch Delphi (Visser-Bochane, Gerrits, Reijneveld, & Van der Schans, 2016). We hope that this is something that we can all agree on and if so, will provide further incentive for taking this forward

**Barriers to agreement**

To understand why it has been so difficult to reach consensus about terminology, we need to recognise the following barriers to agreement:

1. There is no clear dividing line between normality and disorder. The field has attempted to make such a distinction on the basis of the profile of language impairment in relation to other abilities, but this approach is not evidence-based and has arguably led to an unfair allocation of resources.
2. Current classifications tend to use information from a mixture of different levels of description: (i) information about the severity and type of presenting problems with language ; (ii) putative risk factors, such as brain damage, a genetic syndrome or hearing loss; and (iii) co-occurring problems in non-language domains. Implicit in this approach is an attempt to distinguish problems arising from different causes, but causation is often complex and multifactorial, may involve cultural and environmental factors, and is often unknown.
3. Within the domain of language, children's difficulties do not neatly segregate into subtypes, and there may be overlap between problems in speech, language and communication.
4. Terminology can serve different purposes; there may be situations when it needs to be highly precise and others where broader groupings are more useful.

**A. Identifying children requiring additional specialist referral**

***Statement 2****: The term* ***'language disorder'*** *is proposed for children who are likely to have language problems enduring into middle childhood and beyond, with a significant impact on everyday social interactions and educational progress.*

This use of the term 'language disorder' provides consistency with both of the two major classification systems, ICD-11 (forthcoming) and DSM5. Note that disorder is **not** defined in terms of a mismatch between language and nonverbal skills.

This statement clarifies that we recommend using **prognosis** rather than cognitive profile to identify children with language disorder, i.e. those who have language difficulties that lead to significant functional impairment and are unlikely to resolve without specialist help.

We would not include as language disordered children whose problems could be ameliorated by educational interventions delivered in mainstream classrooms, or those who would be likely to outgrow their difficulties.

We have shifted from ‘impairment’ to ‘disorder’, but at the same time placed emphasis on poor prognosis as an important defining feature of language disorder (see also Statement 3). There were several reasons for this change:

Some panel members explicitly stated a preference for ‘disorder’ over ‘impairment’. They felt that it indicated a problem that needed taking seriously, in parallel with other neurodevelopmental disorders (autism spectrum disorder, developmental co-ordination disorder, attention deficit hyperactivity disorder). They also noted benefits that would arise from adopting terminology that is compatible with the two main diagnostic systems (DSM5 and ICD11).

Those who disliked ‘disorder’ gave a range of reasons. It was seen as too ‘medical’, placing the problem ‘inside the child’ (when it might be due to the environment), and raising the spectre of the discredited delay/disorder distinction. We hope that by using what we know about prognosis to define disorder, we can counteract these problems.

One point on which there has been agreement from the outset of CATALISE is that some children have language problems that are severe and persistent enough to create long-term functional difficulties, in daily communication and/or educational attainment. These are the children that ‘disorder’ is intended to refer to: the same ‘tier 3’ cases that we referred to in the background document for CATALISE 1, which is reproduced in Figure 1 here:


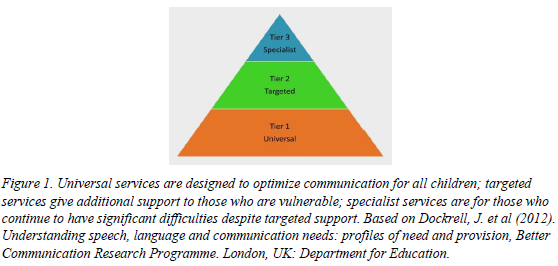


Our focus on these cases should reassure those panel members who are concerned that we might end up over-identifying as ‘disordered’ children who might grow out of their problems or who have limited language skills because of lack of exposure to the language of instruction. Specifically we are saying that those who have mild or transient difficulties that are likely to respond well to targeted educational interventions should **not** be identified as language disordered. The requirement of functional impairment and indicators of poor prognosis means that language disorder would not be identified in a child who had a poor language test score but was otherwise giving no cause for concern.

The boundary between language disorder and typical development is necessarily fuzzy, but we do have relevant information from several longitudinal studies that can help determine prognosis. Accordingly, we have added the next item with the aim of being more specific about what information can be used to make this decision.

We would welcome further input from those panel members who are familiar with the literature on longitudinal studies: we are aware that many of you have been involved in the key studies, so are optimistic you will be able to advise on this.

***Statement 3:*** *In general, poor receptive language and language problems that affect a range of functions are indicators of poor prognosis, especially in a school-aged child. (NB If the panel agrees with this approach, this statement will be elaborated further).*

If there is agreement that we should aim to use prognosis in this way, then we will do a more detailed review of the literature, with the help of the panel, to provide more specific recommendations. The list below is incomplete, but gives an idea of the kind of evidence available. Preliminary indicators of poor prognosis, supported by research are:

1. In toddlers who are late talkers, notably poor comprehension as well as small vocabulary, or failure to use gesture to communicate
2. In older children, poor receptive language (Beitchman et al., 1996) (Clark et al., 2007)
3. Language impairment affecting a range of different functions (D. V. M Bishop & Edmundson, 1987)
4. Inability to retell even a simple story from a picture book at 4 years of age (D. V. M Bishop & Edmundson, 1987)
5. Persistence of language impairment from 4 yr to age 5 or 6 years (Stothard, Snowling, Bishop, Chipchase, & Kaplan, 1998)
6. Poor scores on measures of grammar (Catts, Fey, Tomblin, & Zhang, 2002)
7. Lower nonverbal IQ (Catts et al., 2002; Johnson, Beitchman, & Brownlie, 2010)

In contrast, there is generally a good prognosis for restricted problems with expressive phonology in pre-schoolers (Beitchman et al., 1996). Botting et al found that for 7-yr-olds, family income and maternal IQ not predictive of outcome at 11 yr.

*The specific prognostic factors listed here are a preliminary list to given an idea of what this might look like – there is a relevant literature from longitudinal studies – indeed many panel members have been involved in such studies and we hope you will be able to contribute thoughts to make this more specific, assuming we will go ahead with this conceptualisation. It would be helpful to also be able to specify prognosis for different age bands.

This completely supersedes what was originally statement 1 , which emphasised using information about nature and extent of language difficulties as main basis for diagnosis. That statement had 76% agreement, and 13% disagreement, but numerous questions were raised, which have prompted this reconceptualization.

**B. Risk factors and co-occurring problems**

The principal change here has been the drawing of a distinction between differentiating factors and risk factors – as defined below in statements 6 and 11.

***Statement 4****. The traditional view of exclusionary factors for diagnosis can lead to denial of services to children who might benefit from intervention.*

In some cases it has led to a situation where children are only regarded as meriting intervention if they have remarkably pure language disorder.

This is derived from original statement 11, which achieved 87% agreement.

***Statement 5****. Some children may have language needs in the classroom because their first or home language is not the language used in the classroom, and they have had insufficient exposure to the language of instruction to be fully fluent in English. This should not be regarded as language disorder, unless there is evidence that the child also has difficulties in the home language.*

This would include, for instance, the case of a child with English as an additional language, whose vocabulary may be weak because they have limited exposure to the language used in the classroom. They don’t have a disorder, and should respond well to extra support at school. This would also include hearing-impaired children whose native language is Sign. Where a spoken language is the child’s second language, they are likely to struggle with it, but they would not be regarded as language-disordered unless their native competence in Sign was also compromised.

Many panel members were confused by the earlier statements that made a distinction between ‘language knowledge’ and other aspects of language. That was intended to capture the idea that the child might have weaknesses that reflected inadequate experience with a specific language. That term is now dropped, and it is hoped that this wording will be clearer: the aim is to be clear that a child may obtain a low score on a language test but this does not necessarily mean that the child has any kind of disorder.

***Statement 6****. Rather than using exclusionary criteria in the definition of language disorder, we recommend specifying the presence of any* ***differentiating conditions****. Differentiating conditions are typically biomedical conditions in which language disorder is part of a more complex pattern of impairments that may indicate a different intervention pathway.*

***Statement 7****. Differentiating conditions include autism spectrum disorder, acquired aphasia after brain injury, neurodegenerative conditions, genetic conditions such as Down syndrome, and oral language difficulties associated with hearing loss in children who do not use Sign.*

This is new

***Statement 8****. We recommend referring to ‘Language disorder associated with X’, where there is a differentiating condition, X, as specified above.*

Statement 8 is derived from original statement 9, which gave fairly polarised responses: 62% agreement, 8% neutral and 30% disagreement. We have made two changes that we hope will allow for greater consensus. The original statement used the term ‘secondary to’, and some panel members stated a preference for ‘associated with’, which is now used. Also, we are now much more specific about what the differentiating conditions (X) could be, and are clear that social disadvantage, or unfamiliarity with the language of instruction, should not be used in this way (this was a concern for some respondents).

***Statement 9.*** *In some contexts, particularly when doing research on underlying causes, it makes sense to distinguish cases of language disorder who do not have any differentiating conditions.*

This is a modified version of original item 12, which achieved 91% agreement. Panel members noted, however, that there could be value in research on aetiology that took a more dimensional perspective.

It was also noted there was a need for research on interventions that included broader ‘real world’ samples; nevertheless, to do such research we would still need to be able to distinguish those with and without differentiating factors if we wanted to find out whether an intervention developed for a child with no biomedical history was also effective, say, for children an acquired language disorder or for those with ASD.

***Statement 10****. The term* ***developmental language disorder*** *is proposed to encompass cases of language disorder with no differentiating factors.*

“Developmental language disorder” is consistent with ICD11, though our definition does not use cognitive referencing. It is also compatible with ‘Language disorder’ as used in DSM5.

'Developmental' in this context refers to the fact that the condition emerges in the course of development, rather than being acquired or associated with a known genetic cause. It has been noted that the term 'developmental' can become less useful, or even confusing, as individuals grow older. The solution may be to drop the 'developmental' part of the term in adulthood – this is how this situation is typically handled in the case of (developmental) dyslexia, where affected adults usually refer to themselves as ‘dyslexic’.

There was no clear favourite in the terminology that we proposed previously in original statements 13, 14, and 15. “Primary language impairment” achieved 39% approve, 44% disapprove; “Specific language impairment” achieved 30% approve, 59% disapprove; “Developmental language impairment” achieved 41% approve and 44% disapprove. The marginal preference for ‘developmental’ coupled with the fact that we could achieve consistency with DSM5 and ICD11 by use of ‘language disorder’ has led us to this recommendation.

Although many panel members preferred this term, some objections to the term ‘developmental’ were encountered. Most often there was concern about the inappropriateness of the term for older adolescents and adults: we suggest a workaround in the accompanying text above. Elsewhere some panel members interpreted ‘developmental’ with specific meanings that were not intended: e.g., that this was something that the child might ‘grow out of’, or – quite the converse - that a developmental problem meant that the child would be unable to develop language. Some panel members thought this term would be hard for parents to understand – though similar objections were made for other alternatives that were offered, namely ‘primary’ and ‘specific’.

***Statement 11.*** *Differentiating conditions are distinguished from* ***risk factors****, which are biological or environmental factors that are statistically associated with language disorder, but whose causal relationship to the language problems is unclear or partial, and which are not usually regarded as indicating a different intervention pathway.*

This is new and intended to specify factors that may be important to note, but which should not be used in an exclusionary sense.

***Statement 12****. Risk factors include male gender, a family history of language impairment, poverty, fewere years of parental education, neglect or child abuse, or prenatal/perinatal problems.*

This distinction between differentiating and risk factors is made on the basis of three considerations: (a) how strong the association is between the factor and language disorder; (b) whether the factor typically is associated with additional impairments beyond the language domain; and (c) whether the factor is likely to lead to a distinct intervention pathway. This latter consideration is typically judged on a clinical basis, as we lack good evidence comparing how different groups of children respond to different interventions.

The distinction between differentiating and risk factors is new, but was influenced by some of the comments which were made to the initial diagram showing the three aspects of identification/assessment.

We have added specific mention of poverty, neglect, child abuse and poor parental education, on the basis of panel members’ comments.

***Statement 13. Co-occurring disorders*** *are impairments in other cognitive or behavioural domains that can co-occur with language disorder and may affect prognosis, but whose causal relation to language problems is unclear.*  *These include attentional problems, motor problems, literacy difficulties, speech difficulties, executive impairments, limitations of adaptive behaviour and/or behavioural problems.*

These should be noted, as they may affect prognosis or response to intervention, but they should not be regarded as excluding a diagnosis of language disorder.

***Statement 14****. A child with a language disorder may have a level of nonverbal ability at the low end of the normal range. This does not preclude a diagnosis of language disorder.*

It is now recognised that restricting diagnosis to children with a large discrepancy between nonverbal and verbal ability leads to denial of services to children who could benefit from intervention.

Statements 13 and 14 derive from original statement 16, which had 93% agreement. Some panel members did not like statements that indicated co-occurring problems were common, on the grounds that this information comes from clinical studies, and is not necessarily the case for epidemiological samples. Nevertheless, our focus here is on children who present clinically, and the general view of the panel supported the view that a focus on ‘pure’ cases in the past had been counterproductive.

It was also pointed out that we don’t have much information on how associated factors affect prognosis. If panel members are aware of such information, we could incorporate this in Statement 3.

***Statement 15****. In clinical and educational contexts, allocation of services to children with language disorders should be done according to clinical need. Presence of risk factors or co-occurring disorders should not preclude a diagnosis of developmental language disorder.*

This is based on original item 11, which achieved 87% agreement, 6% neutral and 8% disagreement.

**More specific terminology**

N.B. in the first round of this Delphi, several panel members were concerned that we talked of 'flexible' terminology. We do not mean that people can just pick and choose or use terms loosely, but rather that we may have a nested set of terms, so that we can use a broader label where appropriate, but be more precise where needed. The goal, though, is that individual terms will be clearly specified (as far as our current state of knowledge allows.)

***Statement 16****. Nested within the category of developmental language disorder, more specific terms can be used to pinpoint the principal areas of language difficulty. These would be in addition to, rather than instead of, the term 'developmental language disorder', acting as optional descriptors.*

These more specific terms may be useful for specialist SALTS/SLPs or for researchers, to indicate specific types of problem and to help specify type of intervention required. In some research contexts, it can also be useful to focus on children with a particular type of difficulty, rather than adopting a very broad category of language disorder. Note, however, that there are no clear boundaries between subtypes, and many children have multiple areas of language difficulty. Terminology in this area is likely to change as knowledge advances.

Figure 2 shows how these optional descriptors are subordinate to the broader category of developmental language disorder.

The majority of panellists endorsed original item 4, which was similar to this statement, with only 8% giving moderate or strong disagreement. It was recognised that 'language disorder' was a broad and heterogeneous category and there could be value in giving more specification in some contexts. The few negative comments expressed concern that if additional terms were not clearly and consistently defined we might have a free-for-all. It was also important to be clear that the aim would be to have additional terms as descriptors to accompany 'developmental language disorder', rather than as alternatives – hence some rewording as above.


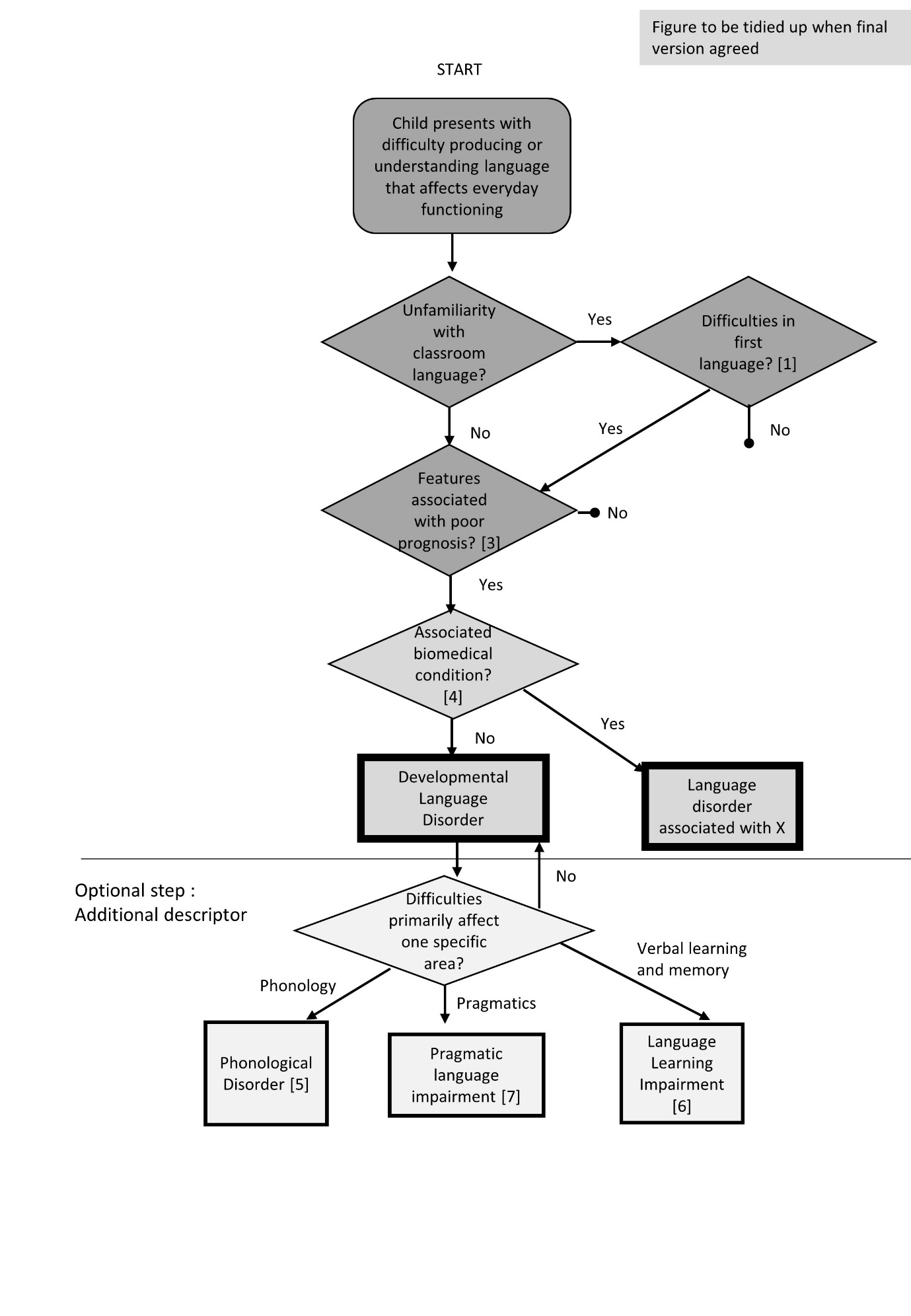


Figure 2: Diagnostic flowchart showing hierarchical relationship between terms

We outline some of the most common terms below in statements 17-20.

***Statement 17****. Where the child’s speech production is characterised by phonological errors, we propose the term* ***Phonological Disorder.***

Differentiating between phonological disorders and other types of speech production problems requires specialist skills. A phonological problem is a difficulty with speech production that is linguistic in origin, rather than one due to motor impairment or physical abnormality of the articulators. It is identified when a child fails to make a speech distinction between sounds that are used to contrast meaning in the language being learned, as when a child says ‘take’ rather than ‘cake’, collapsing ‘t’ and ‘k’. Phonological errors of this kind are normal in early development, but can persist and, when numerous, impair intelligibility of speech. In CATALISE1, the panel agreed that expressive phonological problems should be included under the broader heading of language problems, although when they are not accompanied by other language difficulties, they would lead to a distinct intervention pathway.

***Statement 18.*** *Phonological Disorder is a subset of the category of* ***Speech Sound Disorder****, which is a term in widespread use. This encompasses phonological disorder, but also includes problems with speech production that have their origins in motor or physical problems, rather than having a linguistic basis.*

Statement 17 derives from original statement 7, for which there was 67% agree, 20% neutral and 13% disagree.

Statement 18 has been added to address concerns that structural and motor difficulties with speech had been explicitly excluded. Conceptually, problems with articulation and speech motor programming are speech rather than language problems, and are distinct from phonological difficulties that reflect a failure to learn the phonological categories that are distinctive in the ambient language. However, in practice, these can co-occur, and it is customary to group speech sound disorders together. The new statement 18 recognises this possibility, and relates our classification to the existing literature. As one comment noted “SSD is pretty commonly used and makes no assumption about the causal origins”.

Some commentators mentioned issues around subtyping of speech disorders in children. This is itself a terminological minefield that we are aiming to avoid as far as possible! If panel members agree, we could refer to something like this model from Caroline Bowen’s website (scroll down to the ‘umbrella’ figure) <http://www.speech-language-therapy.com/index.php?option=com_content&view=article&id=45>.

***Statement 19****. For cases of language disorder where the main problems are with pragmatics/social communication and who do not meet diagnostic criteria for Autism Spectrum Disorder, we propose the term* ***Pragmatic Language Impairment.***

This usage is in line with ICD11, which includes pragmatic language impairment (PLI) as a descriptive qualifier within developmental language disorder. There is, however, overlap with the DSM5 category of social (pragmatic) communication disorder (SPCD). There is a helpful summary of the two systems of terminology by Baird and Norbury (2016).

We considered adopting the DSM5 term, but the preference was for PLI for three reasons. First, in DSM5, the motivation for creating a new category of SPCD was evidence that many children had language and communication difficulties characteristic of autism, but without the restricted and repetitive behaviours needed for a diagnosis (Baird & Norbury, 2016). Thus SPCD subsumed some forms of PDDNOS, and is viewed by many as a milder subtype of autism spectrum disorder (ASD). Second, in DSM5, SPCD is seen as a new category of neurodevelopmental disorder, rather than a subgroup within language disorder, whereas we regard it as a subtype of language disorder, compatible with ICD11. This view is supported by a recent study that found that children identified with PLI had similar levels of receptive language as non-autistic children with language disorder, and poorer levels of expressive language than children with high-functioning autism (Gibson, Adams, Lockton, & Green, 2013). Third, perhaps because of its origins in PDDNOS, the label SPCD emphasises social communication, rather than language; in contrast, our focus is on linguistic aspects of impairment. Interventions are being developed that address linguistic as well as social aspects of such communication problems (C. Adams, 2008), and situating PLI as a subset of developmental language disorder should help direct children to appropriate intervention.

This statement is based on original statement 6 for which there was 67% agree, 16% were neutral and 17% disagree. We did encourage people to give their views on specific terminology and we had many useful comments that have helped clarify how to move forward.

We had previously noted options of Pragmatic Language Impairment or Social Communication Disorder (SCD). Several panellists noted that it would be confusing to introduce a new term ‘social communication impairment’ when SCD has already been introduced in DSM5. There was a widespread view that if we were going to have ‘social communication’ we should be compatible with DSM5 and use SCD – or SC(P)D – with p for pragmatic. Views varied on how far ‘social communication’ was intelligible to parents – in general this does seem a point in its favour compared with PLI.

There were arguments put in favour of Pragmatic Language Impairment (PLI), with three panellists specifically referring to Cathy Adams’ framework (C. Adams, 2015). The latest version of the statement (above) is heavily influenced by the recent Baird & Norbury paper that notes that the new ICD11 will use PLI as a descriptor of a subtype of language disorder – this is in line with the hierarchical model proposed here.

Other points expressed were concerns that SCD would be interpreted as meaning social skills problems and/or autistic features, and be used only for children who narrowly fell short of meeting criteria for ASD – points picked up in the text above. There were also concerns that the definition of SCD implies that children with structural language difficulties are excluded (though that does depend on exactly how the definition is interpreted), whereas in practice the literature suggests difficulties with pragmatics are seldom as ‘pure’ as the SCD definition implies (Gibson et al., 2013). This could create diagnostic difficulties for a child with substantial pragmatic impairment who also had milder problems in other language domains, and it may make it harder to access language intervention.

***Statement 20****. The majority of children with developmental language disorder have problems in processes of verbal learning and memory. For these children we propose the term* ***Language Learning Impairment.***

Our understanding of these disorders comes from studies of children designated as cases of specific language impairment (SLI), who generally have problems in retaining sequences of sounds or words over a short delay (verbal short-term memory), forming representations of grammatical constructions in long-term memory, learning associations between words and meaning, or learning statistical patterns in sequential input (Archibald & Gathercole, 2006; D. V M. Bishop, North, & Donlan, 1996; Campbell, Dollaghan, Needleman, & Janosky, 1997; Conti-Ramsden, 2003; Ellis Weismer, 1996; Gillam, Cowan, & Day, 1995; Leonard et al., 2007; Lum, Conti-Ramsden, Page, & Ullman, 2011; Lum & Zarafa, 2010; Montgomery, 2002). These children are likely to require different types of intervention from those with relatively pure phonological disorders or pragmatic language impairment. Their language limitations are also different from those due to poor hearing or auditory discrimination, to lack of knowledge due to unfamiliarity with the ambient language (see Statement xx), or to restricted language input (as in the case of a child whose limited vocabulary reflects lack of exposure to a wider range of words).

In our current state of knowledge, problems in the language learning domain can be evidenced by tests of verbal memory and learning and/or assessment of response to intervention.

We anticipate that the subgroup of LLI is likely to encompass most children who were previously identified as having SLI, but without the connotations of an IQ discrepancy.

This is an elaboration of original item 5, for which there was 62% agree, 19% neutral, and 19% disagree. Among the verbal comments the following points were made

(NB the supportive references cited in the text above are a rather ad hoc collection just to illustrate the range of relevant literature: we would aim to provide a more thorough characterisation if there is agreement on this category).

a) Several respondents commented that surely all language disorders involve 'language learning' problems. The text is now expanded to explain more clearly that poor language skills can arise for a range of reasons, and the term LLI is intended to distinguish cases where the language learning process itself appears to be impaired.

b) Some panellists felt the term LLI would cause confusion with similar terminology that had been used primarily in the USA, to refer to children who had additional learning needs in the context of reading/spelling problems who might not have a functional communication impairment. Others felt there was potential for confusion with 2nd language learning. We have not been able to find clear examples of these usages in the research literature. A search on 'web of science' gave 8 papers prior to 2014 with 'language learning impairment' in the title. These studies did not specifically require evidence of poor learning and memory, but the selection criteria for the LLI group appeared similar to those typically used to identify SLI: low scores on language tests coupled with normal range nonverbal ability and no exclusionary factors, in children who had had speech-language intervention. The small number of papers using the term, and the fact that the way it was used is close (though not identical) to that proposed here, does suggest it may be possible to use this without causing confusion. But we appreciate that there may be other usages in some areas within the school system, and if panellists have examples of these, please do let us have them.

c) There was concern that we don't have good enough assessments to distinguish language learning from other types of problem, and that reliance on a one-off memory test would be insufficient. Assessments based on response-to-intervention are favoured by some panellists, but we are not in a position to be very directive on this. We could give more specific guidelines, and would welcome suggestions.

Please note, ‘language learning disability’ was suggested by some panel members, but in the UK this would not be acceptable, because 'learning disability' refers to children with intellectual disability. As noted above, an advantage of ‘impairment’ is that LLI is similar enough to SLI that it might allow for a relatively painless transition from one term to the other, at the same time allowing us to lose the unfavourable connotations of SLI.

If panel members are not in favour of this term, please do add comments to let us know

1. whether you would prefer to remove this category altogether (it is optional anyway, but it would then leave us with Phonological Disorder and PLI rather stranded– unless we ditched those as well…)
2. Whether you’d like to retain the idea of having a category of this kind – but would prefer another conceptualisation/label for it.

Finally, please note that (as is made clearer below), children do not fit neatly into categories, and there will be children with developmental language disorder who can’t be subclassified this way – either because they have other types of problem, or because they have multiple areas of impairment. Note that the flowchart allows for us to just use the general ‘developmental language disorder’ term in such cases without differentiation.

**Broader terminology**

***Statement 21****. It can be useful to have a broad category for policymakers, because the numbers of children with specific needs in the domain of speech, language and communication has resource implications. The term* ***Speech, Language and Communication Needs (SLCN****), already in use in educational services in the UK, is recommended for this purpose.*

Developmental language disorder can be viewed as a subset within a broad category that covers the whole range of problems affecting speech, language and communication, regardless of nature or putative aetiology.

As shown in Figure 3, this is a very broad category that encompasses children with developmental language disorder (as defined above), but also includes cases where problems have a clear physical basis (e.g. dysarthria), or affect speech fluency or voice. Also included here are children who have needs due to limited familiarity with the language used in the classroom, and those who have communication difficulties as part of autism spectrum disorder.

It is not anticipated that this terminology will be useful for those doing research on the nature or causes of language disorders, nor will it be helpful in explaining a child’s difficulties to parents or in determining a treatment pathway. It could, however, serve a purpose for those who need to plan services, who may need to estimate how many children are likely to require additional support. In addition, it recognises children who have language needs that may require extra help or accommodations in the classroom, even if they do not have a language disorder. These would include those who are shown in pathways terminating in ● in the Flowchart in Figure 2, i.e., children with milder difficulties who should respond well to classroom modification, hearing impaired children who use Sign language, or children who have limited exposure to the ambient language.

This is based on original statement 8. This gave 72% agree, 11% neutral and 17% disagree. The comments made by panellists indicated a wide variety of views ranging from supportive to hostility to incomprehension.

Some comments illustrated the mismatch between those adopting educational vs medical perspectives. So some thought the term ‘needs’ was unhelpful, whereas others liked the fact that it could be used to indicate a problem that was not ‘an internal deficit in the child themselves’ – e.g. the needs of a child who was unfamiliar with the language of instruction are very real, but the child does not have a disorder.

Figure 3: please add comments to this statement if you have thoughts on revising this. We considered adding a dotted line to show cases encompassed by ‘speech sound disorder’ but have so far not done this, as it would make the figure more complex, and SSD is not a major focus.

Figure 3: Depiction of different labels as nested constructs, showing how Developmental Language Disorder is a subset of SLCN, with the descriptors of LLI, PLI and Phonological disorder nested within Developmental Language Disorder.


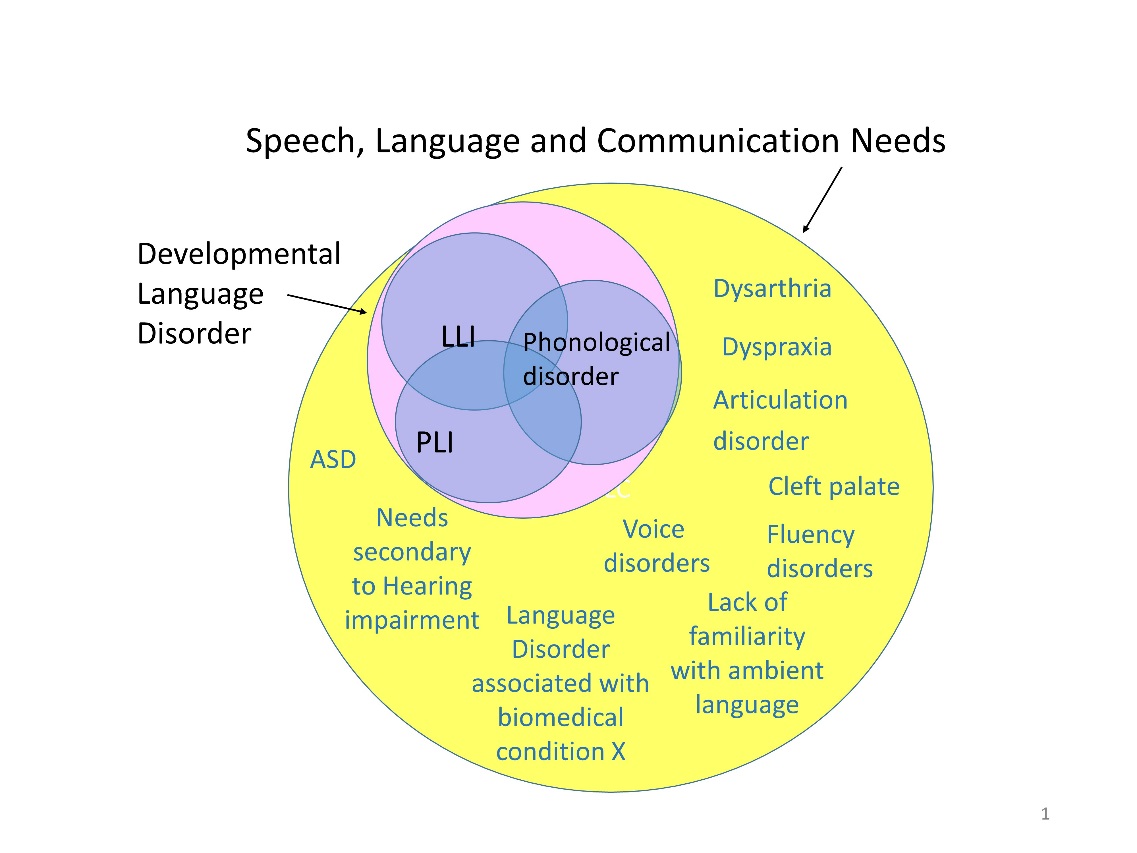


References

Adams, C. (2008). Intervention for children with pragmatic language impairments: frameworks, evidence and diversity. In C. F. Norbury, J. B. Tomblin, & D. V. M. Bishop (Eds.), *Understanding Developmental Language Disorders*. Hove: Psychology Press.

Adams, C. (2015). Assessment and intervention for children with pragmatic language impairment. In D. Hwa-Froelich (Ed.), *Social Communication Development and Disorders*. New York: Psychology Press.

Archibald, L. M. D., & Gathercole, S. E. (2006). Short-term and working memory in specific language impairment. *International Journal of Language and Communication Disorders, 41*, 675-693.

Baird, G., & Norbury, C. F. (2016). Social (pragmatic) communication disorders and autism spectrum disorder. *Archives of Disease in Childhood*. doi: 10.1136/archdischild-2014-306944

Beitchman, J. H., Brownlie, E. B., Inglis, A., Wild, J., Ferguson, B., Schachter, D., . . . Mathews, R. (1996). Seven-year follow-up of speech/language impaired and control children: psychiatric outcome. *Journal of Child Psychology and Psychiatry, 37*, 961-970.

Bishop, D. V. M., & Edmundson, A. (1987). Language-impaired four-year-olds: distinguishing transient from persistent impairment. *Journal of Speech and Hearing Disorders, 52*, 156-173.

Bishop, D. V. M., North, T., & Donlan, C. (1996). Nonword repetition as a behavioural marker for inherited language impairment: evidence from a twin study. *Journal of Child Psychology and Psychiatry, 37*, 391-403.

Campbell, T., Dollaghan, C., Needleman, H., & Janosky, J. (1997). Reducing bias in language assessment: Processing-dependent measures. *Journal of Speech, Language and Hearing Research, 40*, 519-525.

Catts, H. W., Fey, M. E., Tomblin, J. B., & Zhang, X. (2002). A longitudinal investigation of reading outcomes in children with language impairments. *Journal of Speech, Language and Hearing Research, 45*, 1142-1157.

Clark, A., O'Hare, A., Watson, J., Cohen, W., Cowie, H., Elton, R., . . . Seckl, J. (2007). Severe receptive language disorder in childhood-familial aspects and long-term outcomes: results from a Scottish study. *Archives of Disease in Childhood, 92*(7), 614-619.

Conti-Ramsden, G. (2003). Processing and linguistic markers in young children with specific language impairment. *Journal of Speech, Language and Hearing Research, 46*, 1029-1037.

Ellis Weismer, S. (1996). Capacity limitations in working memory: The impact on lexical and morphological learning by children with language impairment. *Topics in Language Disorders, 17*, 33-44.

Gibson, J., Adams, C., Lockton, E., & Green, J. (2013). Social communication disorder outside autism? A diagnostic classification approach to delineating pragmatic language impairment, high functioning autism and specific language impairment. *Journal of Child Psychology and Psychiatry, 54*(11), 1186-1197. doi: 10.1111/jcpp.12079

Gillam, R. B., Cowan, N., & Day, L. S. (1995). Sequential memory in children with and without language impairment. *Journal of Speech and Hearing Research, 38*, 393-402.

Johnson, C. J., Beitchman, J. H., & Brownlie, E. B. (2010). Twenty-year follow-up of children with and without speech-language impairments: family, educational, occupational, and quality of life outcomes. *American Journal of Speech Language Pathology, 19*(1), 51-65.

Leonard, L., Ellis, W., S, Miller, C., Francis, D., Tomblin, J., & Kail, R. (2007). Speed of processing, working memory, and language impairment in children. *Journal of Speech, Language and Hearing Research, 50*(2), 408-428.

Lum, J. A. G., Conti-Ramsden, G., Page, D., & Ullman, M. T. (2011). Working, declarative and procedural memory in specific language impairment. *Cortex, in press*(1-17). doi: 10.1016/j.cortex.2011.06.001

Lum, J. A. G., & Zarafa, M. (2010). Relationship Between Verbal Working Memory and the SCAN-C in Children With Specific Language Impairment. *Language Speech and Hearing Services in Schools, 41*(4), 521-530. doi: 10.1044/0161-1461(2010/09-0035)

Montgomery, J. W. (2002). Understanding the language difficulties of children with specific language impairments: Does verbal working memory matter? *American Journal of Speech-Language Pathology, 11*, 77-91.

Stothard, S. E., Snowling, M. J., Bishop, D. V. M., Chipchase, B. B., & Kaplan, C. A. (1998). Language impaired preschoolers: A follow-up into adolescence. *Journal of Speech, Language and Hearing Research, 41*, 407-418.

Visser-Bochane, M. J., Gerrits, E., Reijneveld, S. A., & Van der Schans, C. P. (2016). Atypical speech and language development: a consensus study on clinical signs in the Netherlands. *International Journal of Language & Communication Disorders*.
