# Supplementary material for: Phase 2 of CATALISE: a multinational and multidisciplinary Delphi consensus study of problems with language development: Terminology
Source: J Child Psychol Psychiatry. 2017 Mar 30;58(10):1068–80. doi: 10.1111/jcpp.12721 (PMC5638113; doi:10.1111/jcpp.12721)
Supplement: Supplementary file 3 — Appendix S3. Relationship between Round 2 statements and final statements reported in Results section. [file JCPP-58-1068-s003.docx]

|  | **CATALISE PHASE 2: Terminology** |  |  |
| --- | --- | --- | --- |
| Final no. | Version in final text | *based on* | Version from Round 2 |
| 1 | *It is important that those working in the field of children's language difficulties use consistent terminology* | 1 | *Achieving agreed terminology for children's language difficulties is a high priority for the field, even if we cannot achieve perfect consensus.* |
| 2 | *The term* ***'language disorder'*** *is proposed for children who are likely to have language problems enduring into middle childhood and beyond, with a significant impact on everyday social interactions or educational progress.* | 2 | *The term* ***'language disorder'*** *is proposed for children who are likely to have language problems enduring into middle childhood and beyond, with a significant impact on everyday social interactions and educational progress.* |
| 3 | *Research evidence indicates that predictors of poor prognosis vary with a child’s age, but in general language problems that affect a range of skills are likely to persist.* | 3 | *In general, poor receptive language and language problems that affect a range of functions are indicators of poor prognosis, especially in a school-aged child.* |
| 4 | *Some children may have language needs because their first or home language is differs from the local language, and they have had insufficient exposure to the language used by the school or community to be fully fluent in it. This should not be regarded as language disorder, unless there is evidence that the child does not have age-appropriate skills in any language.* | 5 | *Some children may have language needs in the classroom because their first or home language is not the language used in the classroom, and they have had insufficient exposure to the language of instruction to be fully fluent in English. This should not be regarded as language disorder, unless there is evidence that the child also has difficulties in the home language.* |
| 5 | *Rather than using exclusionary criteria in the definition of language disorder, we draw a three-fold distinction between differentiating conditions, risk factors and co-occurring conditions.* | 4 | *The traditional view of exclusionary factors for diagnosis can lead to denial of services to children who might benefit from intervention.* |
| 6 | ***Differentiating conditions*** *are biomedical conditions in which language disorder occurs as part of a more complex pattern of impairments. This may indicate a specific intervention pathway. We recommend referring to ‘Language disorder associated with X’, where there is a differentiating condition, X, as specified above.* | 6 | *Rather than using exclusionary criteria in the definition of language disorder, we recommend specifying the presence of any* ***differentiating conditions****. Differentiating conditions are typically biomedical conditions in which language disorder is part of a more complex pattern of impairments that may indicate a different intervention pathway.* |
|  | *(covered in Supplementary information to item 6)* | 7 | *Differentiating conditions include autism spectrum disorder, acquired aphasia after brain injury, neurodegenerative conditions, genetic conditions such as Down syndrome, and oral language difficulties associated with hearing loss in children who do not use Sign.* |
|  | *(Now part of item 6)* | 8 | *We recommend referring to ‘Language disorder associated with X’, where there is a differentiating condition, X, as specified above.* |
|  | *(covered in Supplementary information to item 6)* | 9 | *In some contexts, particularly when doing research on underlying causes, it makes sense to distinguish cases of language disorder who do not have any differentiating conditions.* |
| 7 | *The term* ***Developmental Language Disorder*** *is proposed to refer to cases of language disorder with no known differentiating condition (as defined in Statement 6). Distinguishing these cases is important when doing research on aetiology, and is likely also to have implications for prognosis and intervention.* | 10 | *The term* ***developmental language disorder*** *is proposed to encompass cases of language disorder with no differentiating factors.* |
| 8 | *A child with a language disorder may have a low level of nonverbal ability. This does not preclude a diagnosis of Developmental Language Disorder.* | 14 | *A child with a language disorder may have a level of nonverbal ability at the low end of the normal range. This does not preclude a diagnosis of language disorder.* |
| 9 | ***Co-occurring disorders*** *are impairments in cognitive, motor or behavioural domains that can co-occur with language disorder and may affect pattern of impairment and response to intervention, but whose causal relation to language problems is unclear. These include attentional problems (attention-deficit hyperactivity disorder or ADHD), motor problems (developmental co-ordination disorder or DCD), reading and spelling problems (developmental dyslexia), speech difficulties, auditory processing disorder (APD), executive impairments,  limitations of adaptive behaviour and/or behavioural problems .* | 13 | *Co-occurring disorders are impairments in other cognitive or behavioural domains that can co-occur with language disorder and may affect prognosis, but whose causal relation to language problems is unclear.*  *These include attentional problems, motor problems, literacy difficulties, speech difficulties, executive impairments, limitations of adaptive behaviour and/or behavioural problems.* |
| 10 | ***Risk factors*** *are biological or environmental factors that are statistically associated with language disorder, but whose causal relationship to the language problems is unclear or partial. Risk factors do not exclude a diagnosis of developmental language disorder.* | 11 | *Differentiating conditions are distinguished from* ***risk factors****, which are biological or environmental factors that are statistically associated with language disorder, but whose causal relationship to the language problems is unclear or partial, and which are not usually regarded as indicating a different intervention pathway.* |
|  | *(Covered in supplementary information to Item 10)* | 12 | *Risk factors include male gender, a family history of language impairment, poverty, fewer years of parental education, neglect or child abuse, or prenatal/perinatal problems.* |
| 11 | *Presence of risk factors or co-occurring disorders should be noted, as they may affect management, but they should not preclude a diagnosis of developmental language disorder. In clinical and educational contexts, allocation of specialist services to children with language disorders should be made according to clinical need.* | 15 | *In clinical and educational contexts, allocation of services to children with language disorders should be done according to clinical need. Presence of risk factors or co-occurring disorders should not preclude a diagnosis of developmental language disorder.* |
| 12 | *Developmental Language Disorder is a heterogeneous category that encompasses a wide range of types of difficulty. Nevertheless, it can be helpful for clinicians to pinpoint the principal areas for intervention, and researchers may decide to focus on children with specific characteristics to define more homogeneous samples for study. We suggest here some guidelines for more in-depth analysis of language problems.* | 16 | *Nested within the category of developmental language disorder, more specific terms can be used to pinpoint the principal areas of language difficulty. These would be in addition to, rather than instead of, the term 'developmental language disorder', acting as optional descriptors.* |
|  | *(Revised in Supplementary information to item 12, to treat as descriptor rather than category)* | 17 | *Where the child’s speech production is characterised by phonological errors, we propose the term* ***Phonological Disorder.*** |
|  | *(Supplementary information to item 12)* | 18 | *Phonological Disorder is a subset of the category of* ***Speech Sound Disorder****, which is a term in widespread use. This encompasses phonological disorder, but also includes problems with speech production that have their origins in motor or physical problems, rather than having a linguistic basis.* |
|  | *(Revised in Supplementary information to item 12, to treat as descriptor rather than category)* | 19 | *For cases of language disorder where the main problems are with pragmatics/social communication and who do not meet diagnostic criteria for Autism Spectrum Disorder, we propose the term* ***Pragmatic Language Impairment.*** |
|  | *(Revised in Supplementary information to item 12, to treat as descriptor rather than category)* | 20 | *The majority of children with developmental language disorder have problems in processes of verbal learning and memory. For these children we propose the term* ***Language Learning Impairment.*** |
| 13 | *It can be useful to have a broad category for policymakers, because the numbers of children with specific needs in the domain of speech, language and communication has resource implications. The term* ***Speech, Language and Communication Needs (SLCN****), already in use in educational services in the UK, is recommended for this purpose.* | 21 | *It can be useful to have a broad category for policymakers, because the numbers of children with specific needs in the domain of speech, language and communication has resource implications. The term* ***Speech, Language and Communication Needs (SLCN****), already in use in educational services in the UK, is recommended for this purpose.* |
